# Considering the market share of vaccines against tick-borne encephalitis reported in Austria

**DOI:** 10.1093/jtm/taac027

**Published:** 2022-02-23

**Authors:** Victoria A Jenkins, Bernard Hoet

**Affiliations:** Medical Affairs, Bavarian Nordic AG, 6301 Zug, Switzerland; Medical Affairs, Bavarian Nordic AG, 6301 Zug, Switzerland

## Abstract

Different market share values for TBE vaccines in Austria are reported during the period 2018–2020. However, both data likely cover a significant under-estimation of doses distributed. Thus, for epidemiological evaluations, market share should be used with prudence and only reported when solid whole-market market share can be validated and referenced.

Dear Editors,

We have read the paper ‘*Effectiveness of two doses of tick-borne encephalitis (TBE) vaccine*’ in the *Journal of Travel Medicine*, January 2022 with keen interest.^1^ The paper raises an important topic regarding the effectiveness of TBE vaccination and provides data for an healthcare professional (HCP) to assess the benefit of providing two doses of the TBE vaccine to a traveller to a TBE endemic region for short-term protection, whilst emphasising the importance of the third priming dose for long-term protection.

However, we do have a comment regarding market share reported in this article. In this publication, market share for FSME-IMMUN was reported to be between 84 and 90% for the period 2018–2020, yet the Authors produced no reference as to where these data were obtained. We realise that the market share in the publication are identical to the data we have purchased from IQVIA (a company supplying data on pharmaceutical market dynamics); nevertheless, the IQVIA data only cover ~400 000 doses sold per year in Austria. The in-market sales data from a different provider, Insight Health, based on 550 000–600 000 doses sold annually provides a market share of ~20–23% for Encepur for all 3 years reported, due to the slightly different dataset used. This difference demonstrates that the market share data reported in this publication provides an incomplete and inaccurate picture of the true TBE vaccine distribution in Austria. From internal sources, including direct sales and tender volumes, we understand both providers still cover a significant under-estimation of the number of vaccine doses distributed in Austria.

Our reason for raising this is to do with scientific accuracy of data reporting. This is fundamental as we see, and welcome, more and more publications reporting real world effectiveness for TBE vaccines. When market share is reported, Authors should provide detailed, referenced and reliable data that represent true vaccine administrations in a country.

Regardless, no data exist to allude to any difference in effectiveness between the two Western TBE vaccines, Encepur and FSME-IMMUN.^2^ So, we recognise that it has a great value, from a public health perspective, to report these effectiveness data even without market share, to support the benefit of overall TBE vaccination. By increasing knowledge and awareness about TBE we hope this will lead to a positive increase in vaccine uptake.^3^

## Author Contribution

Both authors contributed equally to data interpretation, writing and review of the manuscript.

## Funding

No funding to disclose.

**Conflict of interest:** VJ and BH are both employees of Bavarian Nordic AG, Switzerland.
